# Embracing the Intersections of Environmental Science, Engineering, and Geosciences to Solve Grand Challenges of the 21st Century

**DOI:** 10.1021/acs.est.3c04795

**Published:** 2023-07-18

**Authors:** Hang Deng, Daniel Giammar, Wei Li, Avner Vengosh

**Affiliations:** † College of Engineering, Peking University, Beijing 100871, China; ‡ McKelvey School of Engineering, Washington University in St. Louis, St. Louis, Missouri 63130, United States; § Key Laboratory of Surficial Geochemistry, Ministry of Education, School of Earth Sciences & Engineering, Nanjing University, Nanjing 210093, China; ∥ Nicholas School of the Environment, Duke University, Durham, North Carolina 27708, United States

**Keywords:** sustainability, climate change, pollution

**Figure 1:**
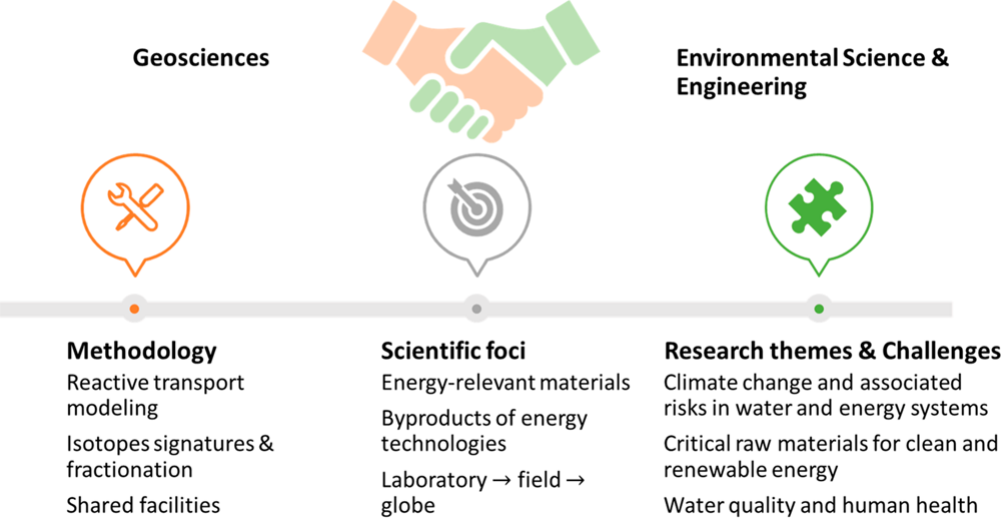
Embracing a geoscience perspective in environmental research.

The U.S. National Academies report on Environmental Engineering for the 21st Century identified five grand challenges of sustainably supplying food, water, and energy; curbing climate change and adapting to its impacts; designing a future without pollution and waste; creating efficient, healthy, resilient cities; and fostering informed decisions and actions.^1^ Addressing many of the grand challenges will require embracing the intersections of environmental science, engineering, and geosciences. These three fields are inherently interdisciplinary, and they naturally intersect with each other (Figure 1). Geosciences, or Earth sciences, study the dynamics of different spheres of Earth. One of its missions is to detect the availability of mineral, water, and fuel resources, for sustainable development,^2,3^ and new research opportunities were identified in “coevolution of life, environment, and climate” and “biogeochemical and water cycles in terrestrial environments and impacts of global change” by the U.S. National Research Council.^2^ Environmental science and engineering focus on the spheres that intersect with human activities. Environmental science puts an emphasis on understanding the migration of naturally occurring and anthropogenic contaminants in the environment and their impact on ecosystem and human health, and environmental engineering centers on developing technologies for water supply, mineral exploration, and environmental remediation to mitigate adverse impacts.^1^

For the environmental science, geoscience, and engineering communities, we posit that it is important to recognize three themes that are particularly intertwined: (1) addressing climate change and the associated risks to our water and energy systems, (2) exploring and supplying critical raw materials that are needed for future clean and renewable energy, and (3) protecting water quality and human health.

Co-design of waste management and energy systems has long been practiced by environmental engineers in pursuing carbon neutrality and mitigating climate change. Additionally, the transition to alternative energy sources and development and implementation of carbon removal technologies have emerged as areas of growth within the environmental science and technology community.^4^ These are research challenges for which a geoscience perspective is valuable for the development of geo-based solutions. Relevant research topics include exploration of alternative energy sources (e.g., geothermal) and critical materials for renewable energy technologies, development of subsurface energy storage systems (e.g., underground storage of hydrogen gas), environmental impact assessment of fossil fuel-based energy systems (e.g., hydraulic fracturing, produced water from oil and gas, and management of coal fly ash), and advancement of geologically based carbon sequestration approaches involving subsurface storage and enhanced rock weathering.^5^

To protect water quality, remediation of groundwater contamination is an important task, which requires the predictive understanding of the fate and transport of emerging anthropogenic pollutants and geogenic contaminants. These are also traditional research priorities of hydrogeology and geochemistry,^6^ including studies specifically focused on the critical zone.^7^ We anticipate that by joining hands with these geoscience branches, the environmental science and engineering communities will generate new opportunities to measure, monitor, and predict the spatial–temporal patterns of various contaminants and thus to accelerate the development and implementation of efficient remediation/treatment technologies.

In accordance with the shared interests in tackling climate change and protecting human health, it is beneficial for the environmental science and engineering communities to expand the research foci from conventional contaminants to include energy-relevant materials or the byproducts of conventional and emerging energy technologies (e.g., CO_2_ and rare earth elements), to fully assess the anthropogenic impacts on our environment for sustainable development. Given the magnitude of the challenges, it is also important to connect scales from the laboratory to not only the field but also the global framework in an effort to devise impactful solutions. One such example is investigation of global cycling of elements that are at the center of emerging energy and transportation technologies.^8^

The intersections of geosciences, environmental science, and engineering are also manifested in increasingly shared research methodology. For instance, shared facilities such as synchrotron beamlines have developed capabilities to handle geological and environmental samples and have seen growing applications in Earth and environmental sciences. Furthermore, some methods that were developed originally in geosciences have been increasingly adopted by the environmental community. One example is reactive transport modeling, which provides physics-based reproduction and prediction of coupled biogeochemical and physical processes. This approach has been widely used in various Earth systems,^9^ and it can also be applied to address the environmental challenges of the 21st century. Reactive transport modeling is particularly useful in tracing the partitioning and fate of chemicals in different environments, integrating laboratory mechanistic observations with truthful representation of the actual environments, and predicting system dynamics at temporal and spatial scales that may otherwise be infeasible. Another example is the use of isotope tracers as environmental indicators that enhance our understanding of the fate and transport of geogenic and anthropogenic pollutants. The advancement of mass spectroscopy has led to the expansion of isotope geochemistry from the analysis of traditional light isotopes (e.g., C, O, and S) to nontraditional metal stable isotopes,^10,11^ opening up entirely new opportunities to determine the sources and pathways of heavy metals in the environment. Investigation of the isotope signatures and isotope fractionation in various environmental processes has become an emerging field that bridges geosciences and environmental science.

*Environmental Science & Technology* has had a history of embracing the intersections of geosciences, environmental science, and engineering, publishing interdisciplinary studies with strong environmental relevance and implications.^12^ A decade ago *Environmental Science & Technology* published work that could be considered as fundamental geochemistry. As the landscape of peer-reviewed journals has expanded, including the launches of *ACS Earth and Space Chemistry* and *ACS ES&T Water*, there are more venues for sharing results that are primarily of geochemical or geological interest. Within this expanded landscape, we see the role of *Environmental Science & Technology* in evaluating and publishing research at the intersection of environmental science, engineering, and geosciences being as important as ever. The themes and tools that we have described are rich with examples of work that yields new scientific insights and has important environmental implications.

The evolution of environmental research is accompanied by integrating knowledge from different disciplines. As Professor James Morgan reflected in the memorial tribute to Professor Werner Stumm, “for protection of aquatic systems, Stumm urged an ecosystem perspective for all aquatic systems”.^13^ We argue that in light of today’s challenges, it is also important to embrace a geoscience perspective in our environmental research.
